# Exploring the biotechnological potential of novel soil-derived *Klebsiella* sp. and *Chryseobacterium* sp. strains using phytate as sole carbon source

**DOI:** 10.3389/fbioe.2024.1426208

**Published:** 2024-06-19

**Authors:** Julieth Maldonado-Pava, Valentina Tapia-Perdomo, Liliana Estupinan-Cardenas, Edinson Puentes-Cala, Genis Andrés Castillo-Villamizar

**Affiliations:** Laboratory of Biocorrosion and Biotechnology, Corporación para la Investigación de la Corrosión (CIC), Piedecuesta, Colombia

**Keywords:** Phytase, phytate hydrolysis, phosphorus metabolism, biocatalyst, microbial diversity, agricultural sustainability, metagenomics

## Abstract

Phosphorus (P) is essential for biological systems, playing a pivotal role in energy metabolism and forming crucial structural components of DNA and RNA. Yet its bioavailable forms are scarce. Phytate, a major form of stored phosphorus in cereals and soils, is poorly bioavailable due to its complex structure. Phytases, enzymes that hydrolyze phytate to release useable phosphorus, are vital in overcoming this limitation and have significant biotechnological applications. This study employed novel method to isolate and characterize bacterial strains capable of metabolizing phytate as the sole carbon and phosphorus source from the Andes mountains soils. Ten strains from the genera *Klebsiella* and Chryseobacterium were isolated, with *Chryseobacterium* sp. CP-77 and *Klebsiella pneumoniae* CP-84 showing specific activities of 3.5 ± 0.4 nkat/mg and 40.8 ± 5 nkat/mg, respectively. Genomic sequencing revealed significant genetic diversity, suggesting CP-77 may represent a novel *Chryseobacterium* species. A fosmid library screening identified several phytase genes, including a 3-phytase in CP-77 and a glucose 1-phosphatase and 3-phytase in CP-84. Phylogenetic analysis confirmed the novelty of these enzymes. These findings highlight the potential of phytase-producing bacteria in sustainable agriculture by enhancing phosphorus bioavailability, reducing reliance on synthetic fertilizers, and contributing to environmental management. This study expands our biotechnological toolkit for microbial phosphorus management and underscores the importance of exploring poorly characterized environments for novel microbial functions. The integration of direct cultivation with metagenomic screening offers robust approaches for discovering microbial biocatalysts, promoting sustainable agricultural practices, and advancing environmental conservation.

## 1 Introduction

Phosphorus (P) is an essential element to all biological systems (Bowler et al., 2010; Kamerlin et al., 2013; Walton et al., 2021). Its significance is underscored by its role as a building block in key molecules like DNA, RNA, and phospholipids. It is also involved in processes such as energy production, enzyme regulation and cellular metabolism (McDowell, 2003; Tiessen, 2008). Among several phosphorus compounds, myo-inositol phosphates emerge as crucial players in eukaryotic cells, contributing to DNA repair, gene expression, osmoregulation and cell signaling (Gonzalez-Uarquin et al., 2020; Su et al., 2023). Myo-inositol hexakisphosphate, chemically known as phytate, serves as the primary phosphorus storage molecule in cereals and grains (Hayes et al., 2000; Liu et al., 2022). Phytate, present in both plant residues and animal feces, accounts for as much as 50% of the organic phosphorus pool in soils (Gerke, 2015; Liu et al., 2022). Despite its natural abundance, the bioavailability of phosphorus in phytate is low to plants and monogastric animals such as poultry and swine (Turner et al., 2002; Menezes-Blackburn et al., 2013). Hence, the use of phosphorus-containing fertilizers and nutritional supplements are required in current agricultural methods, often resulting in environmental eutrophication (Golovan et al., 2001). Phytases, enzymes capable of degrading phytate, are pivotal in unlocking this bound phosphorus. Present in bacteria and fungi, phytases facilitate the utilization of phosphorus from diverse sources holding significant industrial and biotechnological potential (Mullaney and Ullah, 2003; Kumar et al., 2016). Phytases have also shown the release of essential, otherwise unavailable, phytate-bound minerals such as zinc, calcium, iron and magnesium (Handa et al., 2020; Rizwanuddin et al., 2023). This multifunctional aspect of phytate in biological systems underscores the importance of comprehending and leveraging its prospects in microbial biotechnology.

While various studies have highlighted the ability of some bacteria to use phytate as a phosphate source (Shulse et al., 2019; Rizwanuddin et al., 2023), literature addressing its utilization as a carbon source is comparatively scarce (Escobin-Mopera et al., 2012). The discovery of novel bacterial strains capable of utilizing phytate for carbon represents a significant advancement in biotechnology, particularly in agriculture and environmental management. These bacteria can facilitate the release of inorganic phosphate and bound minerals, enhancing soil fertility and plant growth (Kalsi et al., 2016). This is particularly valuable for sustainable agriculture, reducing reliance on resource-intensive synthetic phosphate fertilizers that contribute to environmental pollution. Furthermore, phosphorus fertilizers are unsustainable due to their finite global reserves (Baker et al., 2015). Additionally, they find applications in wastewater treatment, effectively removing excess phosphorus and reducing aquatic eutrophication. Phytate-utilizing bacteria thus contribute to sustainable agriculture and environmental conservation efforts (Kumar et al., 2016).

This study modifies the environmental screening method for bacterial phosphatases described by Castillo Villamizar et al. (2023), integrating direct cultivation of phytate-degrading bacteria with functional screening via a genomic fosmid library. This dual approach efficiently uncovers new strains and species with previously undescribed phytase activities. Direct cultivation isolates phytase-active bacteria, and the fosmid library facilitates the identification of candidate phytase genes. This comprehensive method not only advances our understanding of phosphorus cycling in diverse environments but also enhances the discovery of phytase-producing bacteria, offering significant potential for sustainable agriculture and environmental bioremediation.

## 2 Materials and methods

**FIGURE 1:**
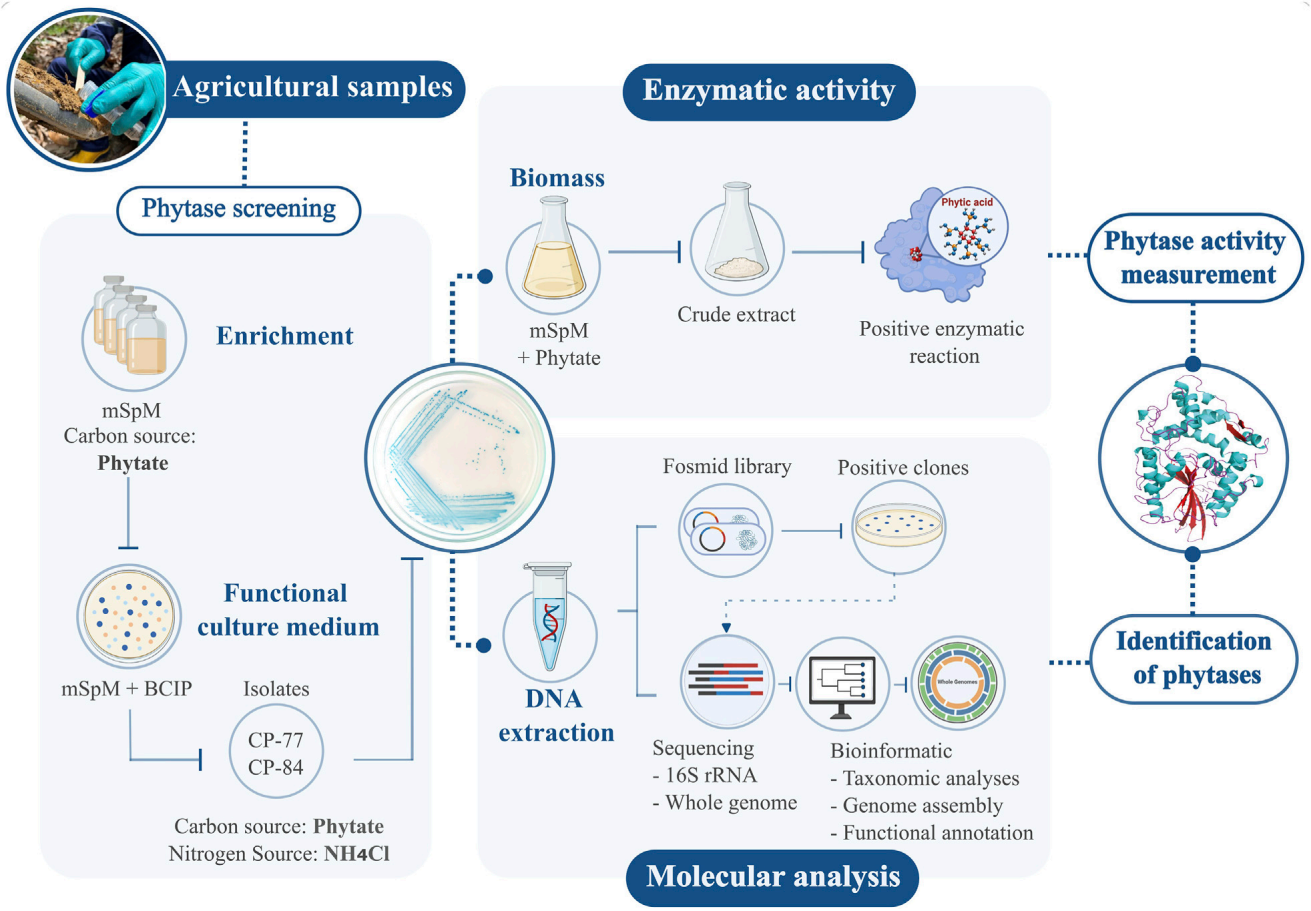
Workflow for detecting phytases in agro-environmental samples. Isolates with positive phytase activity on modified mSpM medium were chosen. Enzymatic activity assays were conducted on selected isolates. Molecular techniques involved constructing a fosmid library, sequencing, and bioinformatic analysis. This integrated approach enabled precise measurement of phytase activity and identification of the responsible genes.

A graphical methodology abstract has been provided in this section to enhance comprehension of the workflow and key procedures employed in this study (Figure 1).

### 2.1 Sample collection and microbial isolation

Soil samples were collected in agricultural sites in Páramo, Santander, Colombia (6° 27′42.0552″N, 73° 9′4.3308″W). Each of the eleven samples underwent processing by resuspending 2 g of soil in 18 mL of sterile PBS. The mixture (2.5 mL) was inoculated into modified Sperber enrichment media (mSpM) containing (g/L): yeast extract 0.5, CaCl_2_ 0.1, MgSO_4_ 0.25, phytic acid 2.5% and 2% v/v glycerol (Castillo Villamizar et al., 2023). Incubation at room temperature for 10 days allowed microbial growth, followed by transfer to fresh media with phytate as the sole carbon source for an additional 10 days. Subsequently, 100 µL of 10^−6^ and 10^−8^ dilutions were plated on solid mSpM medium containing 16 g/L agar-agar and 0.5 g/L NH_4_Cl, replacing yeast extract as the nitrogen source. Phytate served as the exclusive source of phosphorus and carbon. To assess phytate degradation, 5-bromo-4-chloro-3-indolyl phosphate (BCIP) at 25 μg/mL was added as indicator. Colonies exhibiting intense blue coloration, indicative of phytate degradation, were selected for isolation and confirmation of phytate-degrading activity through subculturing on solid mSpM media.

### 2.2 DNA extraction and sequencing

DNA extraction from phytate-degrading isolates followed the protocol by Martín-Platero et al. (2007). DNA concentration and quality were assessed with a NanoPhotometer™ NP80 (IMPLEN, Munich, Germany) and 1% agarose gel electrophoresis. The 16S rRNA gene was sequenced using the 16S Barcoding Kit 1-24 (SQK-16S024), FLO-MIN106 (R9.4.1) flow cells and a Mk1C sequencer by Oxford Nanopore Tech (ONT, Oxford, United Kingdom). *De novo* assembly of sequence reads for each isolate was performed using Flye v. 2.9.1 (Lin et al., 2016), followed by preliminary taxonomic classification via NCBI BLAST (Altschul et al., 1990).

Isolates displaying intense blue coloration in the culture medium, namely, *Chryseobacterium* sp. CP-77 and *Klebsiella* sp. CP-84, were chosen for further genomic and biochemical analysis. Genomic DNA from strains CP-84 and CP-77 was extracted using the Monarch^®^ Genomic DNA Purification Kit (New England Biolabs, Ipswich, MA, United States). Whole genome sequencing was performed with ONT’s Ligation Sequencing Kit (SQK-LSK109) and the Native Barcoding Expansion 1-12 (EXP-NBD104). Additionally, samples were submitted for external sequencing to Novogene Corporation Inc., California, United States, employing the Illumina NovaSeq 6,000 platform (PE150).

### 2.3 Construction of fosmid libraries

A fosmid library was constructed using genomic DNA from strains CP-77 and CP-84. Genomic DNA extraction and end-repair followed manufacturer’s protocols using the DNeasy PowerSoil Pro Kit (QIAGEN, Hilden, Germany) and Copy Control™ Fosmid Production Kit (Epicentre Technologies, Madison, WI, United States), respectively. After purification with AMPure XP beads (Beckman Coulter Inc., Brea, CA, United States), DNA was ligated into the pCC1FOS vector and packaged using MaxPlax Lambda Packaging Extract. Transduction into *Escherichia coli* EPI300-T1R cells was followed by culturing on LB plates with 12.5 μg/mL chloramphenicol at 37°C, with clones preserved at −80°C in 20% glycerol.

Phytase activity screening of the fosmid libraries employed 4-fold diluted solid LB medium supplemented with 2.5 g/L of phytic acid, 12.5 μg/mL chloramphenicol and 25 μg/mL BCIP as indicator. Positive clones underwent culturing in liquid LB medium. Then, fosmid DNA was extracted with the Monarch^®^ Plasmid Miniprep kit (New England Biolabs) and sequenced as described above for bacterial genomes.

### 2.4 Bioinformatics analyses

#### 2.4.1 Phylogenetic analysis based on genomes

Genomes were *de novo* assembled using Unicycler v.0.4.8 (Wick et al., 2017) on the European Galaxy server (The Galaxy Community, 2022). Assembly quality was assessed with QUAST v.5.2.0 (Gurevich et al., 2013), and functional annotation was conducted using Prokka v.1.14.6 (Seemann, 2014). Taxonomic analysis utilized the Genome-to-Genome Distance Calculator (GGDC) and the Type (Strain) Genome Server (TYGS) (Meier-Kolthoff et al., 2013), with genome comparisons performed using the MASH algorithm (Ondov et al., 2016). The 10 closest type strains were identified via the Genome BLAST Distance Phylogeny (GBDP) approach (Meier-Kolthoff et al., 2013). Digital DNA-DNA hybridization (dDDH) values were calculated using default settings of GGDC 4.0 (Meier-Kolthoff et al., 2013; Meier-Kolthoff et al., 2022), and Average Nucleotide Identity (ANI) was determined with OAT v.0.93.1 (Lee et al., 2016). A minimum evolution tree was constructed in FASTME v.2.1.6.1 (Lefort et al., 2015) with a pseudobootstrap of 100 replicates. The resulting tree was visualized using FigTree v.1.4.4 (Rambaut, 2007). Circular comparisons of genomes and plasmids with their closest relatives were visualized using the BLAST Ring Image Generator (BRIG) (Alikhan et al., 2011).

#### 2.4.2 Phytase identification and bioinformatics analysis

Genes annotated as phosphatases underwent comparison with the InterPro database (Paysan-Lafosse et al., 2023) and the Conserved Domains Database (CDD) (Marchler-Bauer et al., 2013) to identify similarities with known phytase families and domains. Phytase sequences were further analyzed for signal peptides, transmembrane helices, and subcellular localization using SignalP v.6 (Teufel et al., 2022), DeepTMHMM v.1.0.24 (Hallgren et al., 2022), and PSORTb v3.0 (Yu et al., 2010), respectively. Three-dimensional protein modeling was conducted with I-TASSER (Zhang, 2008; Roy et al., 2010; Yang et al., 2015), and the models visualized using UCSF ChimeraX (Meng et al., 2023). Related phytases from the Histidine Acid Phosphatase (HAP), Beta Propeller Phytase (BPP) and esterase-like families were retrieved from UniProt and NCBI databases. Sequence alignment was performed using MAFFT v.7 with the maft-homologs function (Katoh et al., 2018). A neighbor-joining phylogenetic tree was constructed based on the JTT matrix model in MEGA11 v.11.0.13 (Stecher et al., 2020; Tamura et al., 2021), incorporating 1,000 bootstrap replicates. The resulting tree was visualized and re-rooted using iTOL v.6.9 (Letunic and Bork, 2024).

### 2.5 Phytase activity assessment

The phytase activity of isolates CP-77 and CP-84 was assessed *in vitro*. Bacteria were cultured in liquid SpM media supplemented with 10 mL/L of a trace elements solution (pH 5.5; containing in g/L: EDTA 5, FeCl_3_ 0.83, ZnSO_4_ 0.178, CuSO_4_ 0.019, H_3_BO_3_ 0.01, Na_2_MoO_4_ 0.04, KI 0.001) under agitation at 250 rpm and 37°C. After 24 h, cultures were centrifuged at 5,000 rpm for 30 min to collect biomass, which was then resuspended in 20 mM Tris-HCl buffer (pH 7) at a 3:1 ratio of buffer to wet weight of biomass. Cell lysis was conducted in 2 mL microcentrifuge tubes filled to one-third of the volume with a mix of glass beads (2.85–3.45 mm, 0.5 mm, and 0.3 mm) with four freeze-thaw cycles at −20°C and room temperature. This was followed by six cycles of homogenization at 4,350 rpm for 1 min each using a BeadBug 6 homogenizer (Benchmark Scientific, Sayreville, NJ, United States), with cooling on ice for 30 s between cycles to prevent overheating. The crude lysate was stored at −80°C for further analyses. Protein concentration was determined using the Bradford method (1976) with bovine serum albumin (BSA) as the standard. Phytase activity was assessed following Greiner’s (2004) protocol, using 1 mM sodium phytate as substrate. The reaction was conducted for 30 min at 37°C. Measurement of the liberated phosphorus was performed according to the ammonium molybdate method (Heinonen and Lahti, 1981) modified by Greiner (2004) at A355 without citric acid addition. Specific activity was expressed in nkatal/mg of protein.

## 3 Results

### 3.1 Identification and characterization of phytase-producing isolates

A modified medium with phytate as the sole carbon and phosphorus source yielded ten strains exhibiting phytase activity on mSpM plates. 16S ribosomal RNA gene sequencing revealed that the isolates belonged to the genera *Chryseobacterium* and *Klebsiella* (Supplementary Figure S1, Supplementary Table S1). Isolates CP-77, CP-78 and CP-87 showed nucleotide identities above 99% to *Chryseobacterium* sp. strain AG844 (CP143637.1). Isolate CP-79 closely resembled *Chryseobacterium* sp. YU-SS-B-43 (KF640081.1), showcasing diversity within the *Chryseobacterium* isolates. Conversely, isolates CP-82 and CP-83 exhibited high sequence identity to *Klebsiella oxytoca* strains FDAARGOS_500 (CP033844.1) and SRY435 (CP138718.1), respectively. Furthermore, isolates CP-80, CP-85 and CP-86, were closely related to different strains of *K. pneumoniae*, including isolate CP-84 to strain KP18-2113 (CP082029.1), underscoring the potential in phosphorus mobilization.

Phytase activity was observed in the crude extracts of both *Chryseobacterium* sp. CP-77 and *Klebsiella pneumoniae* CP-84 (Table 1). The isolates were cultured in liquid mSpM medium with phytate as the sole carbon and phosphate source. In both instances, inoculation into liquid LB medium yielded biomass production without concurrent phytase activity, suggesting that the expression of genes in phytate degradation is likely regulated by inducible gene expression mechanisms.

**TABLE 1:** Phytase Activity in Crude Extracts of *Chryseobacterium* sp. CP-77 and *K*. *pneumoniae* CP-84.

Crude extract	Total protein (mg)	Total activity (nkatal)	Specific activity (nkatal/mg ± SD)
*Chryseobacterium* sp. CP-77	5.9	20.6	3.5 ± 0.4
*K. pneumoniae* CP-84	36	1,468.8	40.8 ± 5

### 3.2 Genome analysis and identification of candidate genes

To deepen our understanding of the genetic basis of phytate degradation and its potential biotechnological applications, we conducted genome analyses on isolates *Chryseobacterium* sp. CP-77 and *Klebsiella* sp. CP-84. These isolates demonstrated rapid growth and intense blue color development on mSpM plates, indicative of phytate transformation. The analysis involved whole-genome-sequencing using a hybrid approach, combining long nanopore reads with short reads from Illumina sequencing. This method resulted in closed genome assemblies for both isolates (Table 2, Supplementary Figures S2, S3) and enabled analyses such as Average Nucleotide Identity (ANI) and digital DNA-DNA hybridization (dDDH).

**TABLE 2:** Comparative Genomic Analysis of *Chryseobacterium* sp. CP-77 and *Klebsiella* sp. CP-84.

Features	*Chryseobacterium* sp. CP-77	*Klebsiella* sp. CP-84
Genome Size (bp)	5,125,303	5,169,325
GC content (%)	36.8	57.7
Coding Sequences (CDS)	4,560	4,722
tRNAs	83	86
rRNAs	18	25
tmRNAs	1	1
ANI^a^ (closest Type Strain)	90.38% (*C*. *cucumeris* GSE06)	99.11% (*K. pneumoniae* DMS 30104)
dDDH4 values^a^	42.1% (CI: 39.6%–44.6%)	94.1% (CI: 92.3%–95.4%)
dDDH6 values^a^	62.9% (CI: 59.6%–66.1%)	88.6% (CI: 85.7%–91.0%)

^a^ The analyses were conducted against the whole genome of the respective closest type strain.

The phylogenetic relationships of *Chryseobacterium* sp. CP-77 and *Klebsiella* sp. CP-84 were investigated using Minimum Evolution (ME) phylogenetic trees (Supplementary Figures S4A, B). These trees incorporated whole-genome sequence data from various species within their respective genera, providing a detailed view of the evolutionary context of each isolate. In the ME tree, *Chryseobacterium* sp. CP-77 formed a distinct lineage within the *Chryseobacterium* genus, closely associated with *Chryseobacterium cucumeris* GSE06. This and the obtained ANI and dDDH values suggests that CP-77 may represent a novel species within the *Chryseobacterium* genus. These phylogenetic distinctions between CP-77 and its closest relatives indicate significant evolutionary divergence, potentially reflecting unique metabolic or ecological traits. For *Klebsiella* sp. CP-84, the phylogenetic analysis places it firmly within the *K. pneumoniae* species complex. It forms a well-supported clade with high bootstrap values, particularly close to *K. pneumoniae* DMS 30104, consistent with ANI and dDDH metrics. This strong genetic similarity suggests that CP-84 is a strain within this often clinically relevant species. After genome assembly and annotation, we identified 22 potential phosphatases in *Chryseobacterium* sp. CP-77 and 70 in *K. pneumoniae* CP-84. Subsequent bioinformatics analyses and the screening of positive clones from the fosmid library unveiled three potential phytases: a 3-phytase (WP_330746005.1) in *Chryseobacterium* sp. CP-77, and a bifunctional glucose-1-phosphatase/inositol phosphatase (glucose 1-phosphatases) (WP_002898698.1) and a 3-phytase (WP_004178993.1) in *K. pneumoniae* CP-84. These sequences underwent further analysis to determine their phylogenetic relationships with known phytases in the NCBI-PDB database. Additionally, detailed NCBI annotation of the *Chryseobacterium* sp. CP-77 genome uncovered an extra protein with phytase-like domains, originally annotated as a hypothetical protein (WP_330745620.1). Domain searches confirmed its association with calcium-dependent phytases and a beta-propeller fold, though it remains uncharacterized. Predictions from SignalP and DeepTMHMM indicated a secretory signal peptide without membrane association, suggesting its role as a soluble enzyme potentially involved in phytate hydrolysis.

**FIGURE 2:**
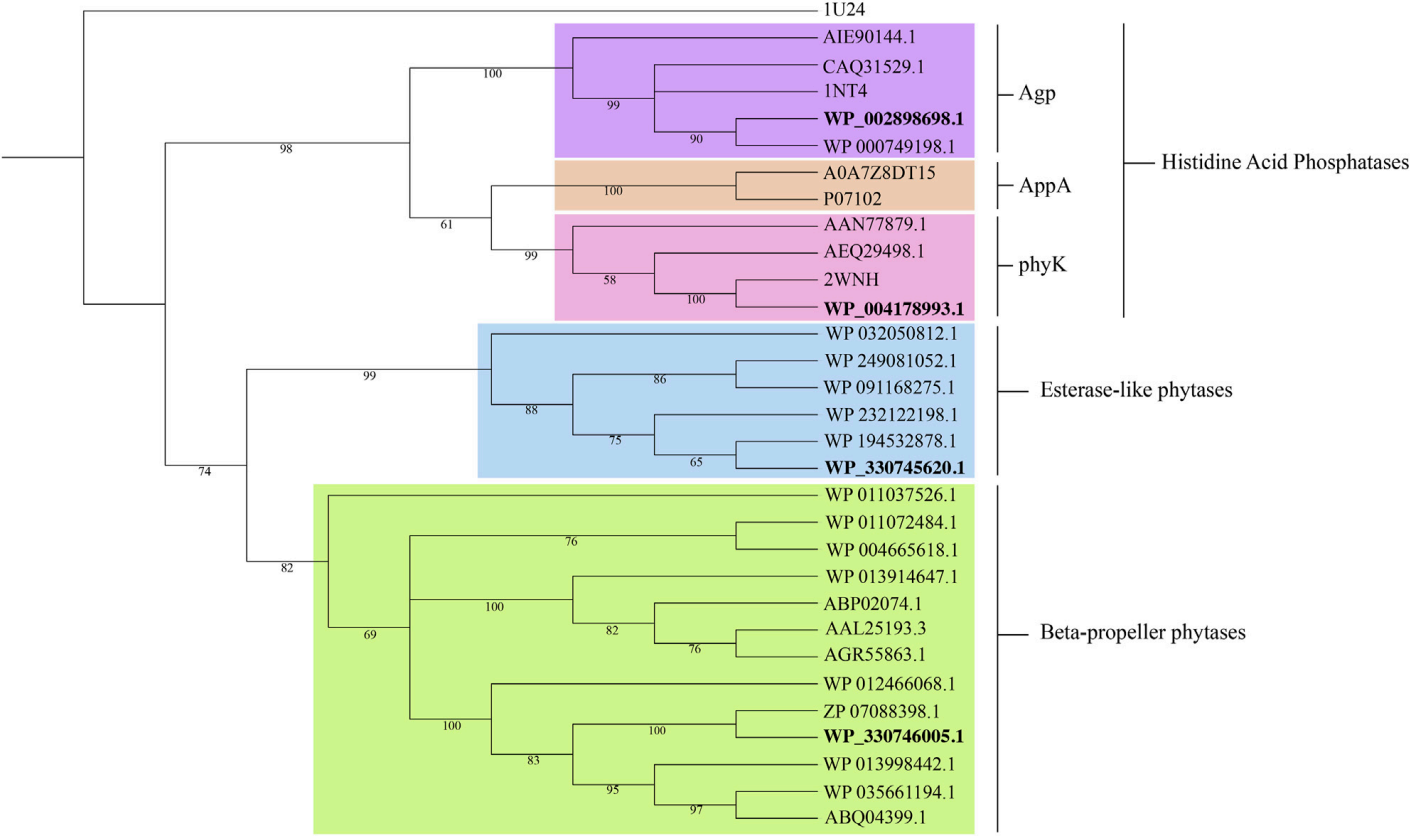
Phylogenetic tree of representative phytases families: Histidine acid phosphatases, beta-propeller phytases and esterase-like phytases. Accession numbers in bold denote the proteins identified in this study. *Selenomonas ruminantium* PTP*-phytase* (1U24) served as outgroup. Bootstrap values from 1,000 replicates are displayed at the respective nodes. Protein sequences were aligned using MAFFT v.7 (Katoh et al., 2018). A neighbor-joining tree was constructed in MEGA11 v.11.0.13 (Stecher et al., 2020; Tamura et al., 2021) and visualized in iTOL v.6.9 (Letunic and Bork, 2024).

The phylogenetic tree reconstruction of phytase families (Figure 2) delineates three principal clades: beta-propeller phytases (BPPs), histidine acid phosphatases (HAPs), and esterase-like phytases. The BPP and esterases-like phytase clades are homogenous, while the HAP clade is diverse, further subdividing into three known subgroups of phytases: Agp-related (glucose 1-phosphatases), AppA-related and PhyK. The positioning of the three phytases from our isolates within this phylogenetic framework is informative. WP_002898698.1 and WP_004178993.1 from *K. pneumoniae* CP-84 clustered within the Agp and phyK-related subgroups, respectively. Meanwhile, WP_330746005.1 from *Chryseobacterium* sp. CP-77 aligned within the classic BPPs, and WP_330745620.1 clustered within the not well-characterized esterase-like phytases.

The putative phytase proteins were modeled using the I-TASSER server (Supplementary Figure S5). Structural modeling of the *Chryseobacterium* 3-phytase revealed similarity to the phytase of *B. amyloliquefaciens* (PDB: 1CVM). Database searches revealed proteins with similar sequences annotated in various *Chryseobacterium* species. The CP-77 3-phytase exhibited 97.97% identity with *C. cucumeri*s (WP_280212031.1) and 31.4% with *B. amyloliquefaciens* (PDB: 1CVM). Structural modeling of the glucose-1-phosphatase from *K. pneumoniae* CP-84, known for its broad substrate specificity and ability to hydrolyze phytate (Wang et al., 2004; Escobin-Mopera et al., 2012), closely aligned with *E. coli*’s G1P (PDB: 1NT4), sharing 79.64% amino acid identity. Additionally, it showed significant identity of 60.39% and 60.63% to *Pantoea agglomerans* (KDA95391.1) and *Pantoea* sp. 3.5.1 (AIE90144.1), respectively. The third protein, 3-phytase from *K. pneumoniae* CP-84 was structurally similar to the phytase from *Klebsiella* sp. ASR1 (PDB: 2WNH), known as PhyK. This enzyme shared high identity with both the previously identified PhyK and the structure from *Klebsiella* sp. ASR1.

## 4 Discussion

This study presents an innovative approach by utilizing a modified minimal medium with phytate as the exclusive source of carbon and phosphorus. This shift from traditional practices, which often incorporate various carbon sources and use phytate merely as a phosphorus supplement (Olstorpe et al., 2009; Castillo Villamizar et al., 2019; Castillo Villamizar et al., 2023), resulted in the successful isolation of ten bacterial strains capable of hydrolyzing the ester bonds of phytate. By restricting available carbon sources to phytate, our study imposed stringent selective pressure, allowing only organisms with the necessary metabolic machinery to thrive. This selective isolation underscores the metabolic adaptability of strains from the *Klebsiella* and *Chryseobacterium* genera, which have evolved to exploit phytate as a crucial phosphorus reservoir. Despite its pathogenicity, *Klebsiella*, a member of the Enterobacteriaceae family, demonstrates remarkable adaptability to diverse environments (Blüher et al., 2017; Morgado et al., 2022). Similarly, *Chryseobacterium*, found in soil and water bodies, exemplifies the metabolic plasticity within the Flavobacteriaceae family (Bernardet et al., 2006). The ability of these strains to thrive in a medium where phytate serves as the sole source of essential nutrients underscores their potential utility in biotechnological applications aimed at phosphorus recovery and recycling.

This method capitalizes on the metabolic diversity of soil microbiomes by utilizing phytate exclusively as both carbon and phosphorus sources, effectively isolating specialized microbial functions. This strategy is particularly valuable for exploring under-characterized regions, such as the Andean mountain agricultural soils (Puentes-Cala et al., 2023; Castillo-Villamizar et al., 2024), highlighting its potential to uncover unique microbial capabilities for sustainable agricultural practices.

Our research represents a significant advancement in understanding phytase activity within the *Chryseobacterium* genus, which has seen rapid expansion from about 10 species in 2005 to 177 according to the DSMZ as of April 2024 (Shimomura et al., 2005; Siddaramappa et al., 2019; Parte et al., 2020). The isolation of *Chryseobacterium* sp. CP-77, a potential new species, sheds light on the metabolic diversity present in poorly studied regions such as the Colombian agricultural soils. The activation of phytase activity in our isolates, particularly in the absence of alternative carbon or phosphorus sources, suggests adaptive gene regulation, likely involving mechanisms akin to the Pho regulon. This regulatory response, triggered by phosphorus scarcity, may also induce enzymes to release myo-inositol from phytate, similar to processes observed in *Bacillus subtili*s (Jha et al., 2019).

While our study successfully isolated and initially characterized phytase-positive strains, the exact identity of the phytase enzyme within *Chryseobacterium* sp. CP-77 remains to be determined. The presence of multiple phosphatase genes and the discovery of additional phytase-like domains within the genome suggest a complex enzymatic system at work. Further research is imperative to elucidate which of these enzymes is the primary contributor to phytase activity. The phylogenetic and structural analyses conducted here highlight the significant potential of the newly identified putative phytases from *Chryseobacterium* sp. CP-77. The modeled structure of the *Chryseobacterium* 3-phytase, showing similarity to the phytase of *B. amyloliquefaciens* (Ha et al., 2000), and high sequence identity with annotated phytases from related *Chryseobacterium* genomes, underscores the evolutionary conservation and potential functionality of this enzyme. However, the absence of documented expression of this phytase in *Chryseobacterium* or its utilization of phytate as a carbon source, suggests untapped metabolic capabilities that require further exploration.

While the discovery of phytase activity in *Chryseobacterium* and the potential new species, CP-77, is promising, it requires detailed physiological and biochemical validation. In contrast, the well-documented phytase capabilities of *K. pneumoniae* (Escobin-Mopera et al., 2012; Houshyar et al., 2023), underscore the utility of integrative approaches like traditional microbiology, genome mining, and metagenomics in uncovering novel biocatalysts. This study successfully identified a 3-phytase in *K. pneumoniae* CP-84, likely responsible for phytase activity, through genome mining of the isolates and a fosmid library screening.

The detection of phytase activity in opportunistic pathogens like *Chryseobacterium* and *Klebsiella* raises intriguing questions regarding their functional roles beyond phosphate metabolism. Existing literature suggests that phytases may contribute to the survival strategies of pathogens like *Xanthomonas campestris*, *E. coli*, and *Candida albicans*. Notably, certain protein tyrosine phosphatases (PTPs), implicated in the pathogenicity of *Mycobacterium tuberculosis*, also exhibit phytase activity (Blüher et al., 2017; Morgado et al., 2022). This hints at a potential link between such catalytic activity and virulence mechanisms in *Klebsiella* and *Chryseobacterium*. In addition, isolates of *K. pneumoniae* and members of the Chryseobacterium genus have been reported to exhibit antibiotic resistance, and their presence in agricultural soils needs to be further addressed (Kirby et al., 2004; Santajit and Indrawattana, 2016; Li et al., 2023). Exploring these connections will be crucial to fully grasp the implications of phytase functions in these bacteria. Moreover, the association of glucose 1-phosphate/phytase and *Klebsiella* 3-phytase with Agp-related phytases suggests their involvement in glucose 1-phosphate metabolism, while *Chryseobacterium* 3-phytase, more closely related to BPP phytases, may participate in broader phosphorus-scavenging pathways. This underscores the multifaceted nature of these enzymes in microbial ecology and pathogenicity. To leverage the potential of these phytases fully, additional experimental validation of their enzymatic activities is imperative. Confirming their functional roles would facilitate the development of technologies aimed at phosphorus recovery and sustainable management practices.

## Data Availability

The datasets presented in this study can be found in online repositories. The names of the repository/repositories and accession number(s) can be found below: https://www.ncbi.nlm.nih.gov/, PRJNA1068642. GenBank codes of 16S ribosomal RNA gene for each isolated: PP593775.1; PP593777.1; PP593800.1; PP593801.1; PP593802.1; PP593803.1; PP593804.1; PP593805.1; PP593806.1; PP593807.1.
