# Supplementary material for: Exploring the biotechnological potential of novel soil-derived Klebsiella sp. and Chryseobacterium sp. strains using phytate as sole carbon source
Source: Front Bioeng Biotechnol. 2024 Jun 19;12:1426208. doi: 10.3389/fbioe.2024.1426208 (PMC11219571; doi:10.3389/fbioe.2024.1426208)
Supplement: Supplementary file 1 [file DataSheet1.docx]

Exploring the Biotechnological Potential of Novel Soil-Derived *Klebsiella* sp. and *Chryseobacterium* sp. strains using phytate as sole carbon source

Julieth Maldonado-Pava^1^†, Valentina Tapia-Perdomo^1^†, Liliana Estupinan-Cardenas^1^, Edinson Puentes-Cala^1^, Genis Andrés Castillo-Villamizar^1*^

^1^Laboratory of Biocorrosion and Biotechnology, Corporación para la Investigación de la Corrosión (CIC), Piedecuesta, Colombia

†These authors contributed equally to this work and share first authorship

# Supplementary Figures and Tables


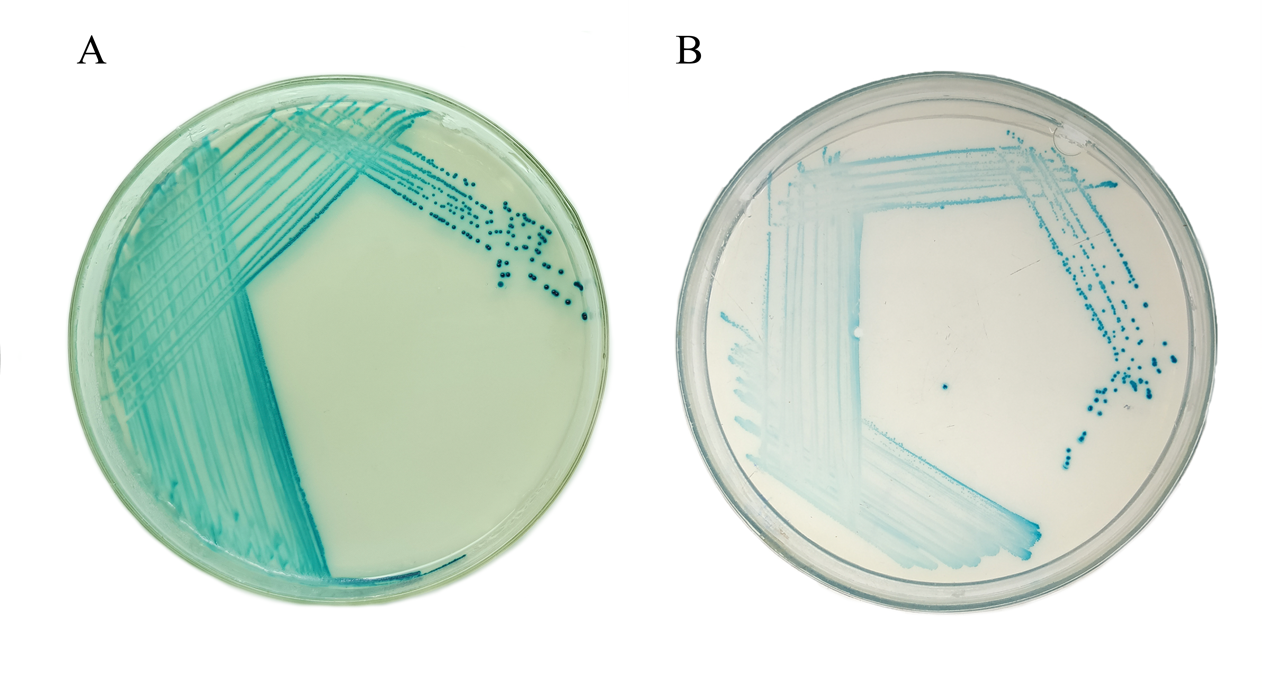


**Supplementary Figure S1.** Screening of phytase activity in Modified Sperber medium. Typical positive activity of the isolates CP-84 (**A**) and CP-77 (**B**).

**Supplementary Table S1.** 16S rRNA-based analysis of strains exhibiting phytase activity on mSpM plates.

| **Most relate organism** | **Isolate ID** | **Identity (%)** | **Coverage (%)** | **GenBank Acc. Number** |  |
| --- | --- | --- | --- | --- | --- |
|  |  |  |  |  |  |
| *Chryseobacterium sp.* strain AG844 | CP-77 | 99.46 | 97 | CP143637.1 |  |
|  | CP-78 | 99.53 | 98 | CP143637.1 |  |
|  | CP-87 | 99.32 | 95 | CP143637.1 |  |
| *Chryseobacterium* sp. YU-SS-B-43 | CP-79 | 99.36 | 93 | KF640081.1 |  |
| *Klebsiella pneumoniae* strain XHKP53 | CP-80 | 96 | 99.53 | CP066891.1 |  |
| *Klebsiella oxytoca* strain FDAARGOS_500 | CP-82 | 98.81 | 97 | CP033844.1 |  |
| *Klebsiella oxytoca* strain SRY435 | CP-83 | 99.34 | 98 | CP138718.1 |  |
| *Klebsiella pneumoniae* strain KP18-2113 | CP-84 | 99.53 | 92 | CP082029.1 |  |
| *Klebsiella pneumoniae subsp. pneumoniae* strain KKP102 | CP-85 | 99.73 | 96 | CP090519.1 |  |
| *Klebsiella pneumoniae subsp. pneumoniae* | CP-86 | 99.64 | 92 | CP090519.1 |  |

**
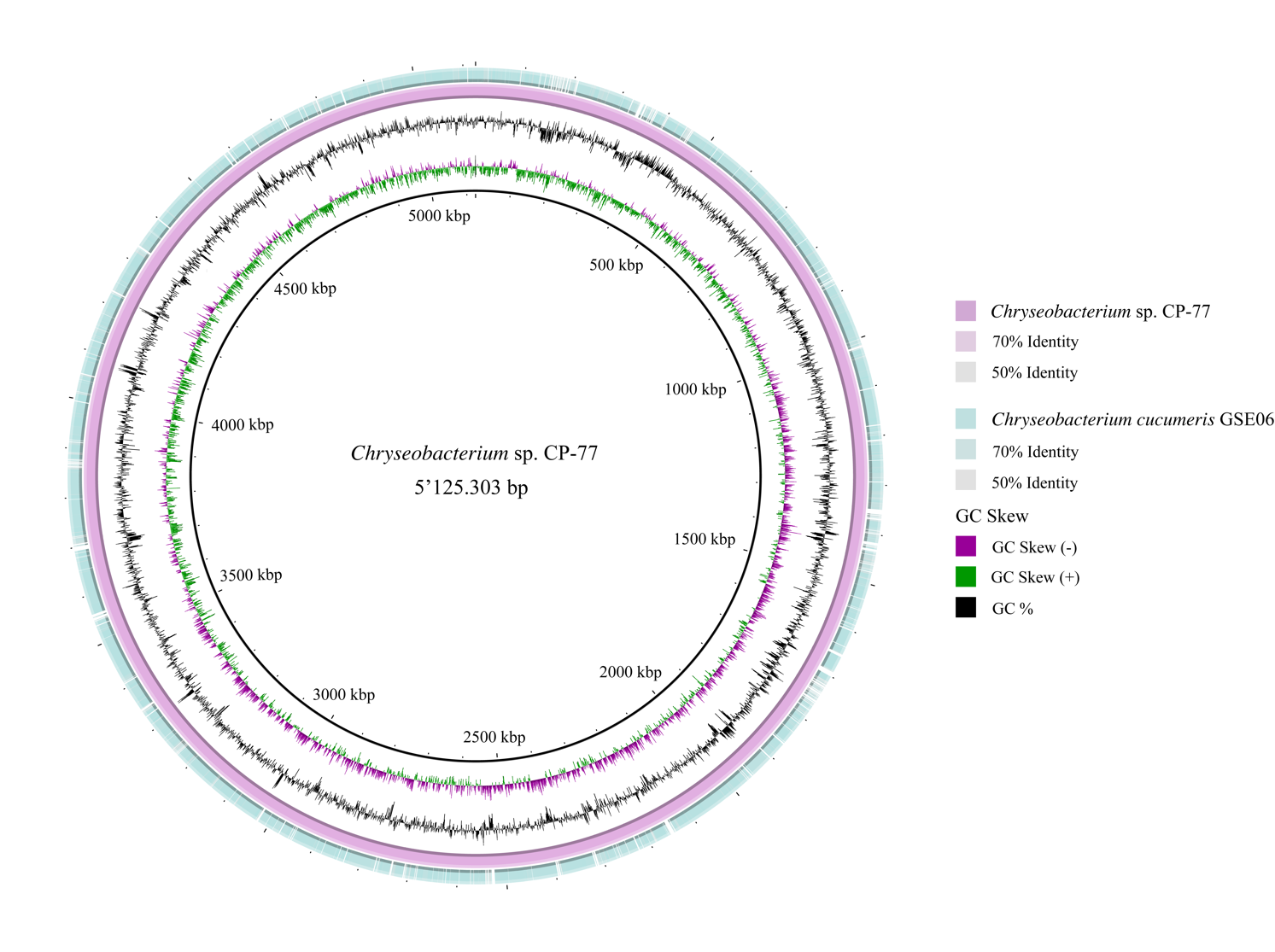
****Supplementary Figure S2.** Genomic map of *Chryseobacterium* sp. CP-77 (CP143637.1) (pink inner ring). The outer concentric ring depicts the chromosome of closest related species, with nucleotide identities relative to CP-77 displayed in color gradients. The GC skew [(G−C)/(G + C)], depicting positive (green) and negative (purple) values, is visualized. The middle circle (black) indicates the percentage of GC content.

**
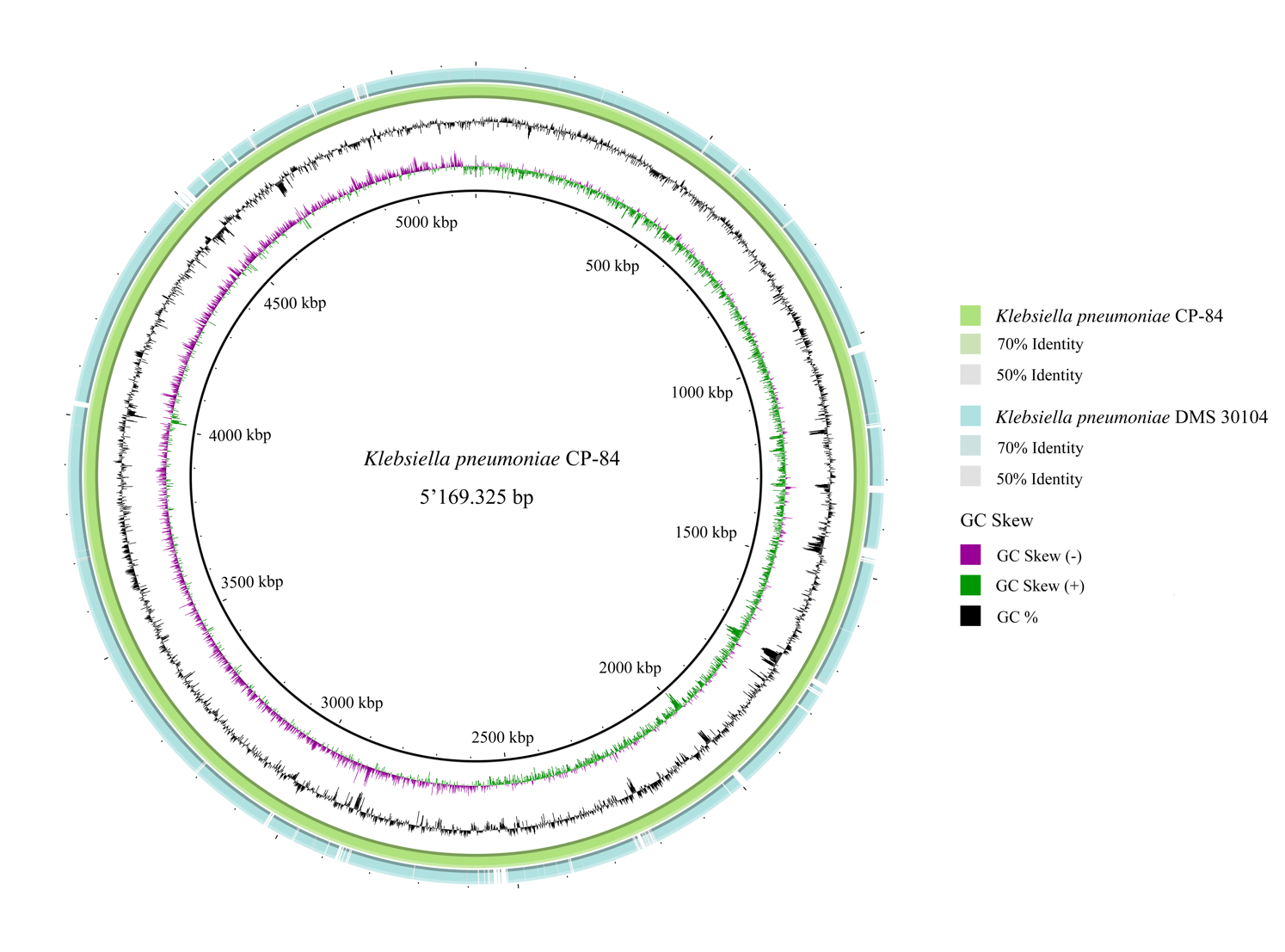
****Supplementary Figure S3.** Genomic map of *Klebsiella pneumoniae* CP-84 (CP143638.1) (green inner ring). The outer concentric ring depicts the chromosome of closest related species aligned, with nucleotide identities relative CP-84 represented in color gradients. The GC skew [(G−C)/(G + C)], represented by positive (green) and negative (purple) values, is visualized. The middle circle (black) indicates the percentage of GC content.

**
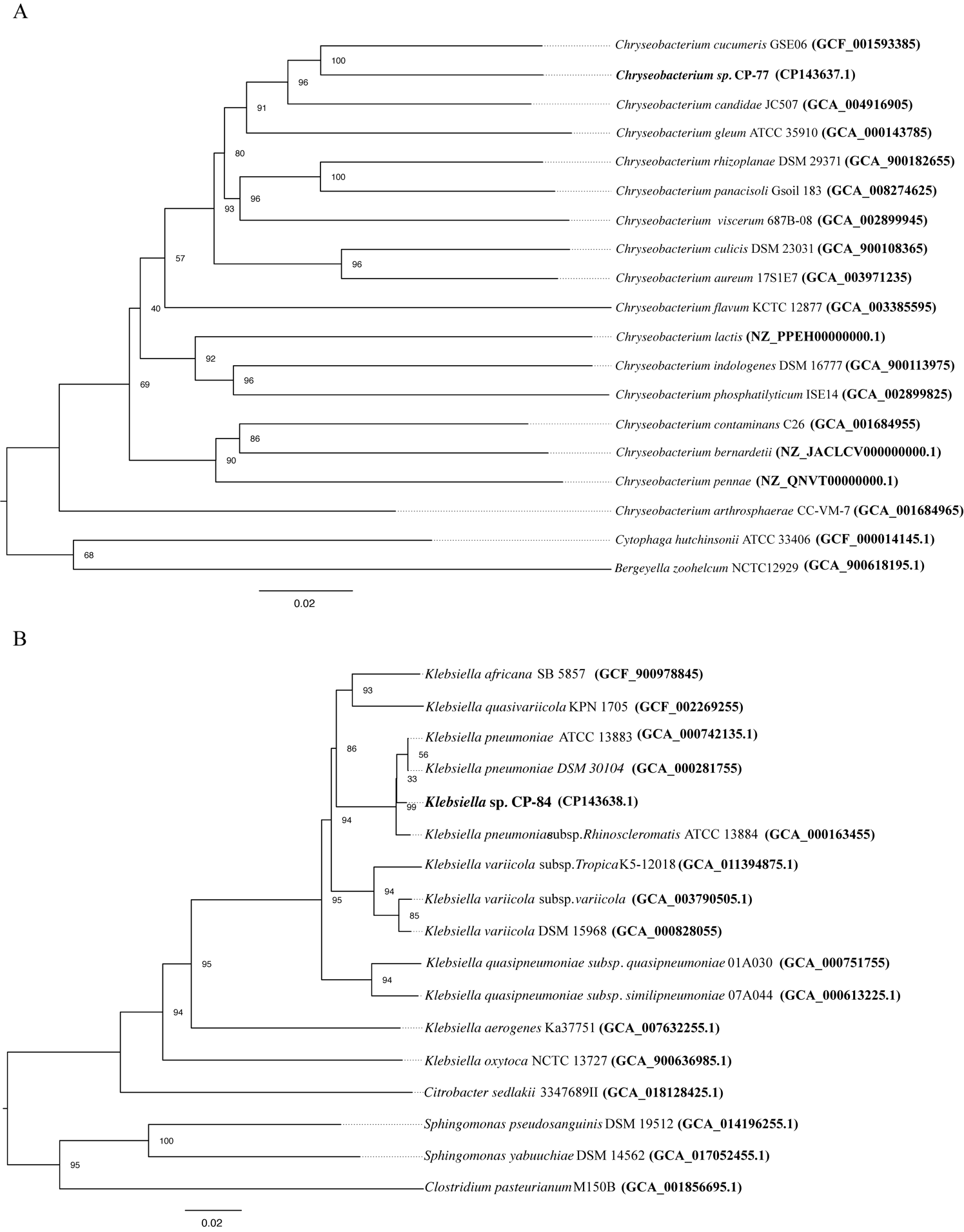
****Supplementary Figure S4.** Minimum evolution tree based on whole genome data showing the phylogenetic placement of *Chryseobacterium* sp. CP-77 (**A**) and *Klebsiella* sp. CP-84 (**B**) relative to type strains of closely related species. Trees were generated using the Type (Strain) Genome Server (TYGS) platform. Branch support was inferred from 100 pseudo-bootstrap replicates.


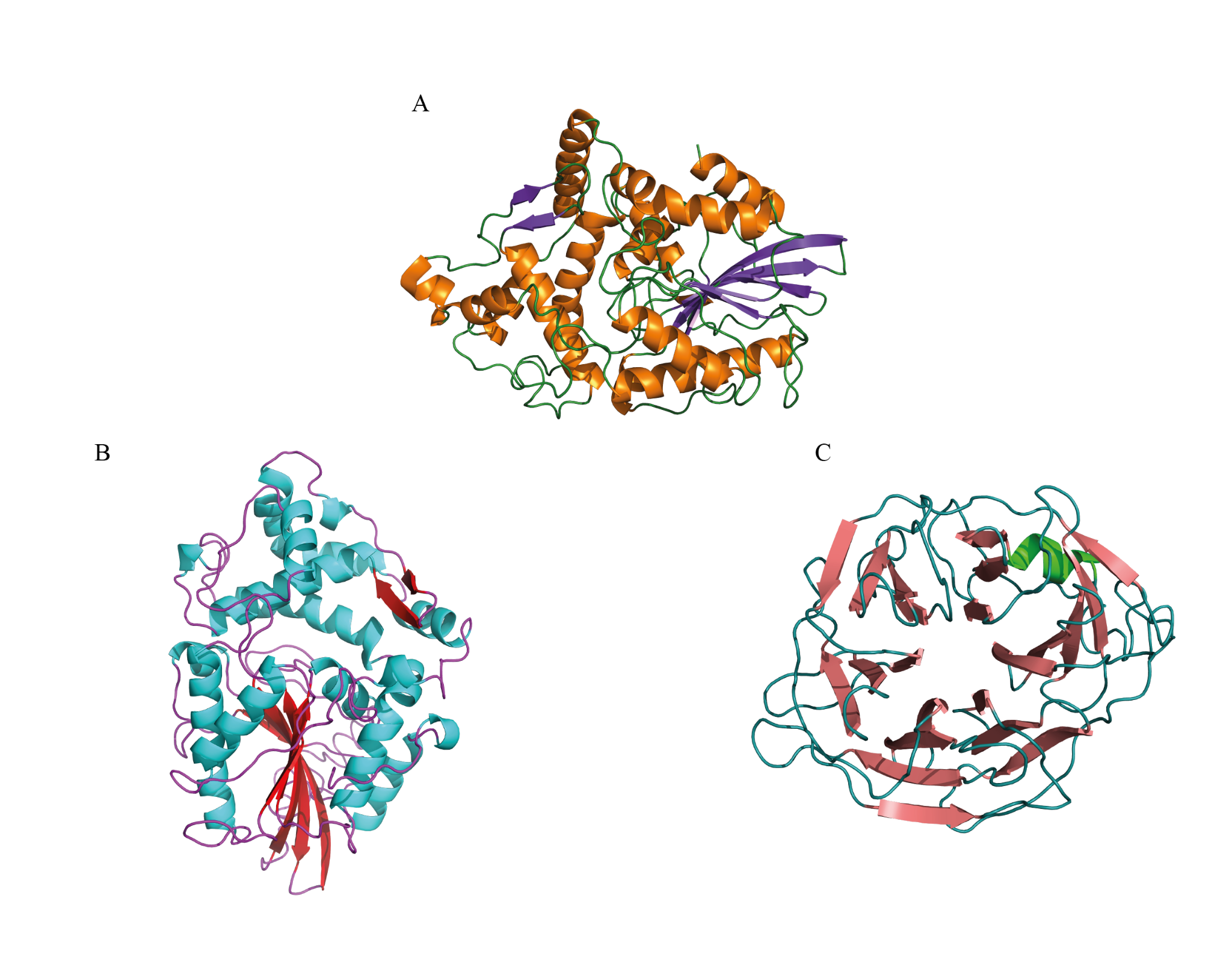


**Supplementary Figure S5**. Three-dimensional prediction models for *Klebsiella*’s, (**A**) Bifunctional glucose-1-phosphatase/inositol phosphatase (WP_002898698.1*),* (**B**) *3-*phytase (WP_004178993.1), and *Chryseobacterium*’s (**C**) 3-phytase (WP_330746005.1) predicted by I-TASSER and visualized with ChimeraX. Obtained C-scores for each model were: -0.46 (A), 0.53 (B) and -0.63(C).
